# Mapping the AI Landscape in Food Science and Engineering: A Bibliometric Analysis Enhanced with Interactive Digital Tools and Company Case Studies

**DOI:** 10.1007/s12393-025-09413-w

**Published:** 2025-07-01

**Authors:** Jordan Pennells, Peter Watkins, Alexander L. Bowler, Nicholas J. Watson, Kai Knoerzer

**Affiliations:** 1 Food Innovation Centre, CSIRO Agriculture and Food, Werribee Victoria, 3030 Australia; 2 School of Food Science and Nutrition, University of Leeds, Leeds, LS2 9 JT UK

**Keywords:** Artificial Intelligence, Food, Bibliometric Analysis, Interactive Dashboard, Large Language Models, Case Studies

## Abstract

The proliferation of research on Artificial Intelligence (AI) in food science and engineering has made it increasingly difficult to synthesise relevant insights effectively. Although AI adoption in the food industry has grown, it lags behind sectors like finance and healthcare due to the complexity of food systems, including high process variability, risk aversion towards novel technologies, and constrained investment appetite. Historically, computational techniques and AI-adjacent technologies like expert systems and empirical modelling have supported food research and development for decades. More recently, AI applications have broadened to include process control, food safety, ingredient and product quality, sensory evaluation, traceability, and supply chain management. In response to the rapid increase in AI-related food science publications – particularly since 2019 – this review introduces tools for dynamically synthesising and exploring this evolving knowledge base. We present an interactive dashboard that integrates a curated dataset of food AI review articles with advanced bibliometric analyses, enabling user-driven exploration of research trends and thematic relationships. Additionally, we demonstrate the use of customised large language model (LLM) tools for targeted literature interrogation, enhancing accessibility for researchers and industry stakeholders. Complementing this academic synthesis, we profile selected industry case studies where AI plays a central role in ingredient discovery, product development, intelligent sorting, and sensory analytics. By combining interactive research tools with real-world case studies, this review offers a comprehensive snapshot of Food AI and begins to bridge the gap between academic research and industry implementation, providing a valuable resource for those seeking both domain-specific knowledge and actionable insights.

**Supplementary Information:**

The online version contains supplementary material available at 10.1007/s12393-025-09413-w.

## Introduction

### Overview

Artificial Intelligence (AI) is reshaping industries worldwide, unlocking new possibilities in efficiency, safety, sustainability, and innovation. From healthcare to finance to agriculture, AI has driven breakthroughs in automation, predictive analytics, and data-driven decision-making. The food industry presents a unique challenge – food systems are deeply complex, highly regulated, and reliant on physical processes, making AI difficult to implement.

Yet, the perception that the food sector has been slow to adopt AI is not entirely true. The industry has long integrated data-driven modelling and computational techniques, from kinetic modelling [1–3] and artificial neural networks (ANNs) [4–6] to hyperspectral imaging [7–9] and computer vision [10–12]. These technologies, while not always explicitly labelled as AI, have played a crucial role in optimising food processing, quality control, and safety monitoring. However, their impact has remained largely confined to research or niche applications until recent advances in computational power, big data availability, and modern machine learning algorithms enabled more scalable and integrated AI applications.

This raises an important question: What exactly is AI in the context of food science and engineering? Traditionally, AI is defined as the replication of human intelligence in machines, enabling them to analyse data, recognise patterns, and make decisions that would typically require human cognition [13]. However, there is no single, universally accepted definition of AI. For instance, the OECD defines AI as “a machine-based system that can, for a given set of human-defined objectives, make predictions, recommendations, or decisions influencing real or virtual environments” [14], while the European Commission describes AI systems as “software (and possibly also hardware) systems designed by humans that, given a complex goal, act in the physical or digital dimension by perceiving their environment… and taking actions - autonomously or semi-autonomously” [15]. Rather than a singular technology, AI should be understood as an evolving ecosystem of computational methods and algorithms that enable diverse applications across the food sector, including food safety monitoring and traceability, quality control, process optimisation, sensory evaluation, and supply chain management.

As AI technologies become more powerful and accessible, the question is no longer whether AI will transform the food industry, but how effectively it will do so to create safer, more sustainable, and more efficient food systems. Yet, critical questions still remain. These include: Is the food industry truly lagging in AI adoption, or has it simply used AI-adjacent technologies under different labels? Is AI driving genuine transformation, or are we witnessing another cycle of technological hype? What technical, economic, or regulatory barriers still hinder its broader adoption in the food industry? As research on AI in food science continues to expand, how can we synthesise this growing body of knowledge in more systematic and innovative ways?

### Scope of the Review

This review addresses the growing need for structured synthesis in the expanding field of AI within the food science and engineering domain. Despite the volume of review articles available, there is limited capacity to explore this landscape systematically or interactively. To overcome this limitation, this review article introduces a comprehensive and methodologically novel approach. Rather than use a conventional approach, we combine systematic bibliometric analysis with digital tools designed to dynamically explore and synthesise existing knowledge. Specifically, we perform a systematic bibliometric analysis of AI-focused review articles, examining the evolution of key research themes and technological trends. Advanced bibliometric methods, including keyword co-occurrence networks, thematic mapping, topic modelling, and document similarity analyses, are leveraged to create a data-driven overview of AI research trajectories and thematic developments in food science. To address the challenge of effectively synthesising an increasingly large and complex literature, we introduce two complementary digital tools: (1) an interactive online dashboard app that provides dynamic visualisations and user-directed exploration of bibliometric data; and (2) customised large language model (LLM) tools designed for user-driven interrogation and synthesis of research content from the compiled corpus of review articles.

These tools are intended to support a wide range of user, including food researchers seeking to identify trends in application areas, industry stakeholders evaluating the maturity of specific AI applications, and AI practitioners seeking details on specific algorithms or approaches taken for specific problems. For instance, a researcher exploring AI methods for detecting damage in soft fruit may use the review to identify commonly used sensors, algorithm types, and data sources for similar applications, or an industry professional assessing predictive maintenance strategies can examine the types of AI techniques reported in peer-reviewed literature and their associated outcomes. By enhancing the transparency, accessibility, and interactivity of the literature, this review provides a novel framework for understanding the current state and future directions of food AI research.

Beyond mapping the research landscape, this review integrates an industry-oriented perspective through selected case studies of companies that apply AI technologies as a central part of their product or service offerings. These examples illustrate how AI is being used in practice for tasks such as ingredient discovery, intelligent food sorting, sensory analytics, product development, and operational optimisation.

Through the combination of bibliometric analysis, interactive tools, and real-world case studies, this review offers a unique and practical resource for researchers and industry professionals aiming to navigate the evolution of AI in food science and identify opportunities for future research and technological advancement.

### The Evolution of AI in Food Science

AI has undergone multiple phases of growth, stagnation, and resurgence since its formal conceptualisation in 1956 [16]. Early AI systems, such as symbolic AI [17] and expert systems, laid the foundation for rule-based decision-making [18]. Expert systems emerged as one of the earliest AI applications in food science, with first reports of systems developed for the food industry dating back to the mid-1980 s [19], related to kinematic modelling for process control [20]. However, these systems were rigid and difficult to scale, requiring human-defined rules that struggled to handle uncertainty and real-world variability, such is the case in dynamic food contexts.

Another early AI-driven innovation in food science was the artificial or electronic nose (e-nose), first proposed in 1982 [21] and developed in the early 1990 s as a biomimetic system to analyse volatile compounds and detect food quality issues [22, 23]. By the mid to late 1990 s, commercial e-nose devices were being sold for food freshness evaluation, spoilage detection, and aroma profiling [24]​. The integration of AI techniques enabled pattern recognition and odour classification, making e-nose technology particularly valuable in coffee aroma evaluation, meat quality assessment, and non-invasive quality control.

The emergence of machine learning in the late 1990 s and early 2000 s marked a shift from rule-based AI to data-driven learning systems, enabling AI to make predictions based on patterns rather than predefined rules. ANNs – an early form of deep learning – and fuzzy logic models gained prominence in food process modelling and quality assessment, such as food grading, safety, and quality checks [25]. However, computational limitations restricted their widespread use at this time.

By the 2010 s, the rise of big data, computational power, and deep learning accelerated AI adoption in food science [26]. AI-powered computer vision systems, initially explored in the 1990 s, became a widely used, non-destructive tool for real-time food quality inspection, defect detection, and quality grading​. Convolutional neural networks (CNNs) enabled more precise and scalable image analysis, enhancing automated sorting and food processing operations.

In the present decade, deep learning and generative AI are driving new advancements for AI in Food. Models such as AlphaFold have revolutionised protein structure prediction, accelerating ingredient discovery and food formulation​. LLMs like ChatGPT are being applied to consumer sentiment analysis, personalised nutrition, and AI-driven product recommendations. Generative adversarial networks (GANs) are being explored for synthetic food image generation, hyperspectral data augmentation, and sensory evaluation modelling​. These innovations are transforming how food products are developed, marketed, and optimised for consumer preferences.

**Fig. 1:**
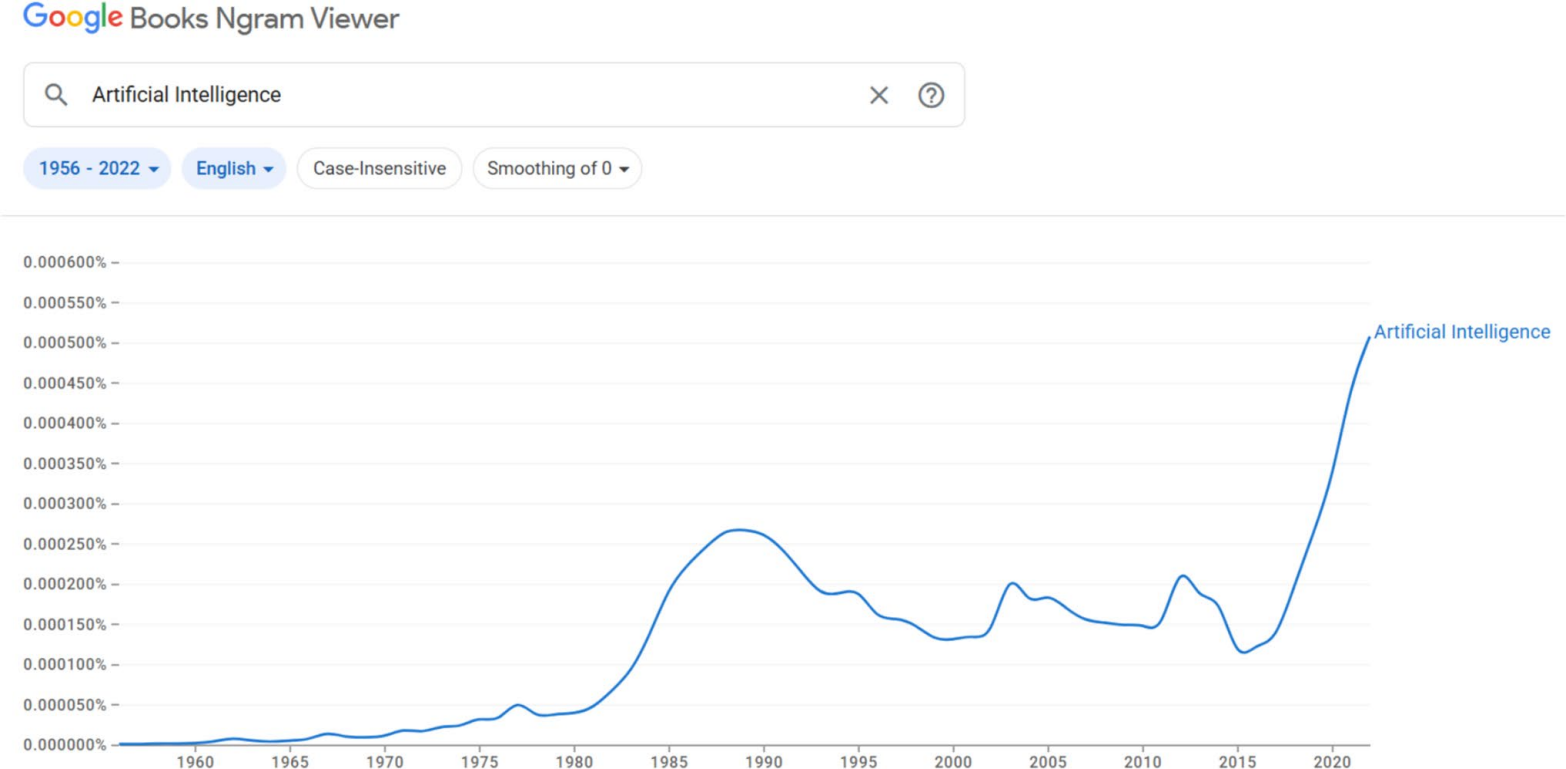
Frequency of the term “Artificial Intelligence” in English-language books from 1956 (origin of AI term) to 2022 (via Google Books Ngram Viewer)

As AI has evolved from rule-based expert systems to modern deep learning architectures and generative models, its influence has expanded dramatically across both academic research and commercial domains. This growth is not limited to technical literature but is also evident in broader sociocultural discourse, as illustrated in Fig. 1, which shows the usage trajectory of the term “Artificial Intelligence” in published books over time. The sharp increase after 2015 outpaces all previous interest peaks aligns with the era of deep learning breakthroughs and AI’s transition from theoretical development to widespread adoption in various industries, including food technology.

## Bibliometric Analysis of Food AI Reviews

### Background

The increasing prominence of AI in food science is reflected in the growing number of research publications exploring its applications across the industry. As AI continues to advance, researchers and industry professionals rely on review articles to synthesise the vast body of knowledge and provide structured insights into how AI is transforming the industry from farm to fork. These reviews highlight key technological trends, emerging innovations, and the challenges associated with AI adoption in food systems.

Given the increasing number of AI-related reviews in food science, bibliometric analysis serves as a valuable tool for mapping research developments, identifying dominant themes, and uncovering gaps in the field. Traditional bibliometric techniques, such as publication trend analysis, co-citation networks, and abstract-based topic modelling, offer a data-driven perspective on how food AI research has evolved over time. However, with the sheer volume of publications available, innovative approaches are needed to enhance accessibility and knowledge synthesis.

To address this, we introduce an interactive online tool designed to complement this bibliometric analysis – the **Food AI Dashboard** app (Online Resource 1) **–** which enables users to dynamically explore publication trends, keyword relationships, and thematic patterns in food AI literature. By combining bibliometric analysis with AI-driven knowledge synthesis, this section aims to provide a comprehensive overview of food AI research while enhancing the way users engage with the literature.

### Bibliographic Analysis Methodology

#### Data Collection

To curate the review article corpus and conduct a comprehensive bibliometric analysis, we utilised the Web of Science (WoS) Core Collection database as of February 2025. Our search strategy targeted review articles related to AI applications in food science by querying Topic fields (title, abstract, author keywords, and Keywords Plus) using “Food” in conjunction with AI-related terms, including “Artificial Intelligence”, “Machine Learning”, “Deep Learning”, “Neural Network”, “Genetic Algorithm”, “Expert System”, “Fuzzy Logic”, “Natural Language Processing”, “Electronic Nose”, “Random Forest”, “Digital Twin”, “Computer Vision”, “Reinforcement Learning”, “Natural Language Processing”, and “Generative AI”. The search was further refined to include only peer-reviewed review articles published in English within the Food Science Technology category. Articles were excluded if they were non-English publications, primary research articles, conference abstracts, retracted papers, or studies not directly related to AI applications in food science.

Each retrieved review article was manually classified based on its primary thematic focus within the food sector. Classification categories included Process Monitoring, Control & Optimisation, Food Safety, Ingredient Quality, Product Quality, Product Development, Sensory Evaluation, Supply Chain Optimisation, Traceability, Personalised Nutrition, Intelligent Packaging, Synthetic Biology, Augmented Reality, Multi-OMICS, and Food Security. The full set of review articles that met the inclusion criteria (N = 213) were exported in BibTeX format and imported into R for bibliometric analysis.

#### General Analysis

The bibliometric data was analysed using the *bibliometrix* R package to generate a comprehensive summary of the dataset [27]. Descriptive statistics were computed to identify key publication trends, including the most productive authors, countries, institutions, and journals contributing to food AI research. Temporal trends in author productivity were visualised to examine the evolution of research contributions over time. Additionally, co-authorship network analysis was conducted to map collaborative relationships among researchers, providing insights into the structure and interconnectedness of the food AI research community.

#### Frequency Analysis

A keyword frequency analysis was performed to determine the most commonly occurring terms within the dataset. This involved quantifying the occurrences of individual keywords and ranking them based on their frequency. The analysis aimed to reveal dominant research themes, emerging topics, and evolving areas of interest within the food AI literature. The results were visualised using bar charts, which illustrated the most frequently used keywords and their relative prominence. These visualisations provided a high-level overview of the primary research focus areas in food AI, offering a data-driven perspective on the field's thematic structure.

#### Keyword Co-Occurrence Network

A keyword co-occurrence network was constructed to analyse the conceptual structure of food AI research by mapping relationships between frequently occurring keywords in review articles. Keywords were extracted, pre-processed, and standardised to ensure consistency, after which the *biblioNetwork* function from the bibliometrix package was used to compute a co-occurrence matrix, quantifying how often pairs of keywords appeared together within the same articles [27].

The resulting network was visualised using a force-directed graph layout, where the size of each node corresponded to keyword frequency, and edge thickness reflected the strength of association between two keywords. This analysis provided insights into dominant research clusters, identifying core research themes, interdisciplinary connections, and underexplored areas within food AI. Examining the network structure and connection density allowed for the identification of central themes shaping food AI research and the mapping of emerging trends over time.

#### Conceptual Structure Map

To identify key conceptual structures within the dataset, correspondence analysis was performed on the document keywords. Correspondence analysis is a multivariate statistical technique used to examine relationships between categorical variables [28]. In this context, it was applied to explore associations between keywords extracted from the review articles, revealing underlying conceptual linkages in food AI research. By mapping keywords onto a low-dimensional space, this method provided a clearer visualisation of co-occurrence patterns. Keywords positioned closer together indicated stronger thematic relationships, enabling a structured interpretation of how different research topics intersect within the food AI landscape.

#### Thematic Map

A thematic map was generated to identify major research themes and their evolution over time using the *thematicMap* function. This method clusters documents based on keyword co-occurrence patterns, providing insights into the structural positioning of research topics within food AI. The thematic map is structured along two key dimensions: centrality (*x*-axis) and density (*y*-axis). Centrality represents the degree of connection between a theme and other topics in the field, indicating its influence within food AI research, while density reflects the internal cohesion and development of a theme, distinguishing well-established research areas from emerging or fragmented topics.

Themes are distributed across four quadrants: motor themes (high centrality, high density), which drive the field and are well-integrated; basic and transversal themes (high centrality, low density), which serve as foundational topics connecting multiple areas; emerging or declining themes (low centrality, low density), which may be developing or losing relevance; and highly developed but isolated themes (low centrality, high density), which are internally cohesive but less connected to the broader research landscape.

#### Topic Modelling

To identify latent themes within the literature, abstracts from the selected review articles were extracted and pre-processed to ensure accurate and meaningful text analysis. The pre-processing steps included converting text to lowercase, removing punctuation and numbers, eliminating stopwords, and stripping extraneous whitespace. A document-term matrix (DTM) was then constructed from the pre-processed abstracts, serving as the foundation for topic modelling.

Latent Dirichlet Allocation (LDA), a generative probabilistic model, was applied to the DTM to uncover underlying topics within the corpus. LDA assumes that documents are composed of multiple topics, each represented as a distribution of words. The number of topics (*k*) was set to six. The resulting topic-term distributions and document-topic distributions were visualised using the *LDAvis* package, allowing for an interactive exploration of the identified themes. To further enhance interpretability, word clouds were generated for each topic, highlighting the most representative terms and providing an intuitive understanding of thematic content within the food AI literature.

#### Document Similarity

To analyse document similarity and identify the most closely related articles within the corpus, a cosine similarity analysis was performed using a Term Frequency-Inverse Document Frequency (TF-IDF) weighted Document-Term Matrix (DTM). TF-IDF is a statistical measure that evaluates the importance of a word within a document relative to a collection of documents, reducing the influence of commonly used terms while emphasising domain-specific terminology [29]. Cosine similarity, which measures the cosine of the angle between two document vectors in a multi-dimensional space, was computed to quantify the degree of similarity between documents. This approach enabled the identification of highly related review articles based on textual content, facilitating a deeper understanding of literature interconnectedness and allowing for the exploration of clusters of closely aligned research.

#### Bipartite Network Analysis

A bipartite network analysis was conducted to examine the relationship between AI methodologies and their applications in food science. Each review article was analysed for the presence of AI-related and food-related terms, which were identified using a predefined dictionary. A document-term matrix was constructed, mapping AI techniques (e.g., machine learning, deep learning, expert systems) to food science domains (e.g., food safety, quality control, supply chain optimisation).

To quantify associations between AI methods and food subdomains, an adjacency matrix was generated, where rows represented AI techniques and columns represented food applications. The strength of connections was determined based on the co-occurrence frequency of terms within the same review articles. The network was visualised using a force-directed graph layout, where node size indicated term frequency, and edge thickness reflected the strength of association.

#### Analysis of Cited Research Articles

To analyse the primary research articles cited within food AI review articles, citation data was extracted and processed to retrieve metadata, including publication year, title, and research topics. The cited references (CR) field from the bibliometric dataset was parsed to extract individual references, which were cleaned and standardised by removing extraneous whitespace and inconsistencies in formatting.

To retrieve DOI (Digital Object Identifier) information, regular expression matching was applied to extract DOIs embedded within references. A set of unique DOIs was then compiled for further metadata retrieval. The OpenAlex API, a publicly available scholarly metadata source, was used to fetch additional bibliographic details, including publication year, article title, and associated research concepts. API queries were performed in batches to optimise request efficiency and prevent rate limitations. The retrieved metadata was then merged with the original reference dataset using DOI-based matching, ensuring accurate alignment between cited references and their corresponding bibliographic records. The final dataset provided insights into the distribution of cited research over time, the most frequently referenced topics, and the thematic connections between review articles and primary research.

### Bibliometric Analysis Results

#### General Analysis

Initially, a manual evaluation was performed to sort the review articles into categories based on their central theme. Table 1 highlights that the top categories for which review articles have been written for AI applications in food include Food Safety (39), Process Monitoring, Control & Optimisation (37), Product Quality (28), and Traceability (23), in addition to General or uncategorised articles (35).

**Table 1:** Categorisation of review articles related to AI and digital technology applications across key categories of the food system

Category	Number of Review Articles
Food Safety	39
Process Monitoring, Control & Optimisation	37
General AI	35
Product Quality	28
Traceability	23
Sensory Evaluation	14
Supply Chain Optimisation	10
Multi-Omics	7
Personalised Nutrition	5
Ingredient Quality	4
Product Development	4
Synthetic Biology	4
Intelligent Packaging	3
Augmented Reality	2
Food Security	1

The bibliometric analysis covered a timespan from 2004 to 2024, encompassing 216 documents sourced from 61 journals, books, and other publications. Although the average document age was only 2.3 years, the average total citations was 40.7, translating to an average of 8.9 citations per year per document. A total of 12,297 references were cited across the documents, at an average of 96 references per document. The analysis identified 454 unique author-provided keywords and included contributions from 666 unique authors. Additionally, 41.41% of the documents involved international co-authorship. The results generated by the *biblioAnalysis* function are presented in full within the Food AI Dashboard app.

**Fig. 2:**
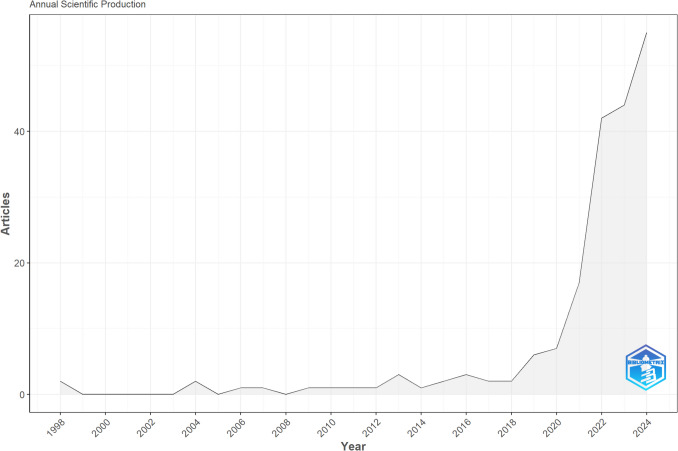
Annual production of scientific review articles from 1998 to 2024 related to food AI

The annual scientific production in Fig. 2 illustrates a significant increase in review article publications from 2004 to 2024, particularly from 2019 onwards. Before 2019, no more than 3 review articles had been published per year, which has changed dramatically as of 2022, where 42 review articles were published. This sharp increase indicates a surge in research attention towards synthesising the research of AI applications in food science. This trend reflects the rapid advancements and adoption of AI technologies more broadly in everyday living and the recognition of AI in the public consciousness.

**Fig. 3:**
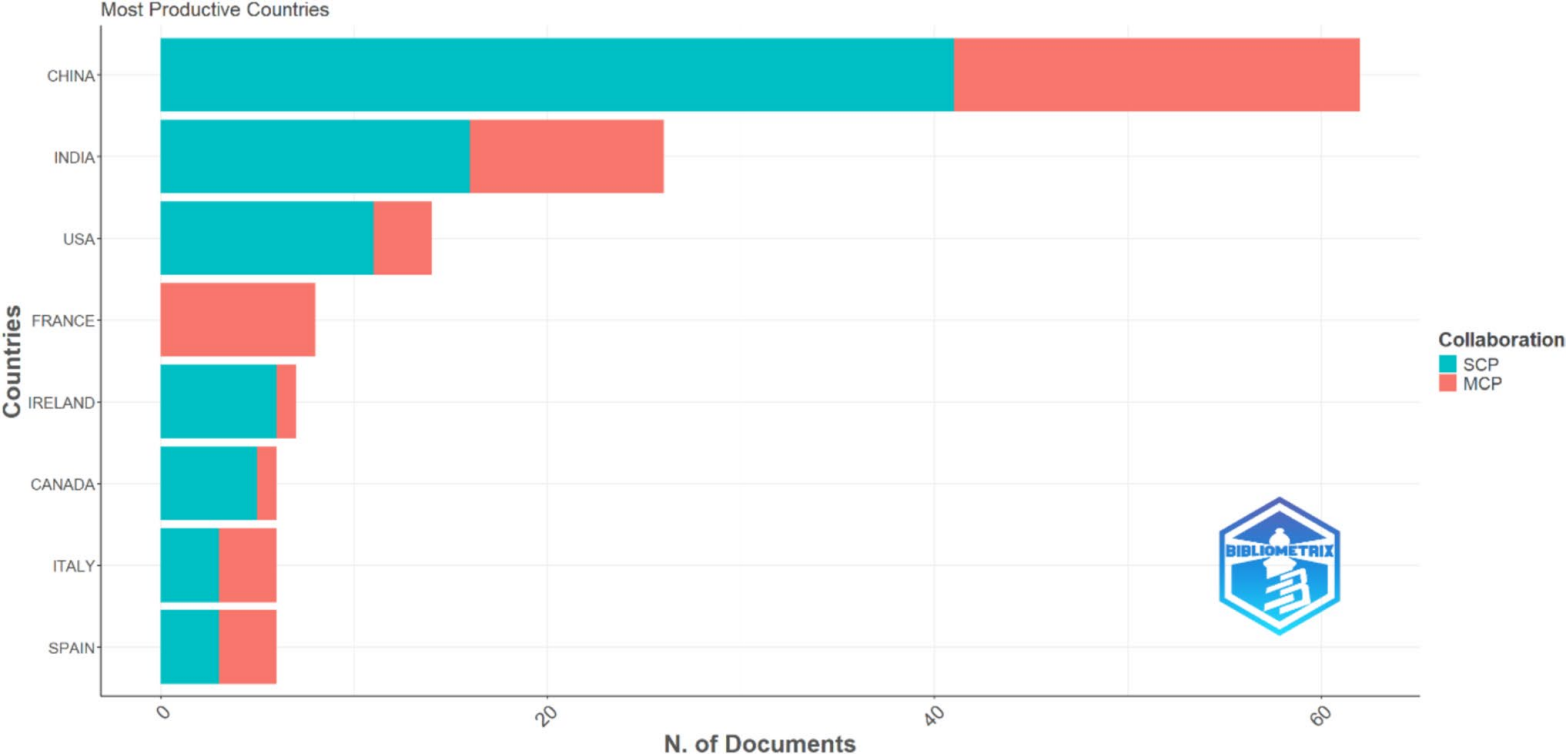
Most productive countries for food AI review publications. SCP: Single Country Publications, MCP: Multiple Country Publications

Figure 3 presents the distribution of food AI review articles based on the author’s country, including their contributions in terms of single-country publications (SCP) and multiple-country publications (MCP). China and India are by far the most productive countries when it comes to publishing review articles on the topic of food AI, with a total of 35 and 18 publications, respectively. French authors have published 6 review articles, all of which are multiple country publications, highlighting their extensive collaboration with international researchers. Researchers from Italy, Malaysia and the US have published 5 review articles, with a balance of domestic and international collaboration.

**Fig. 4:**
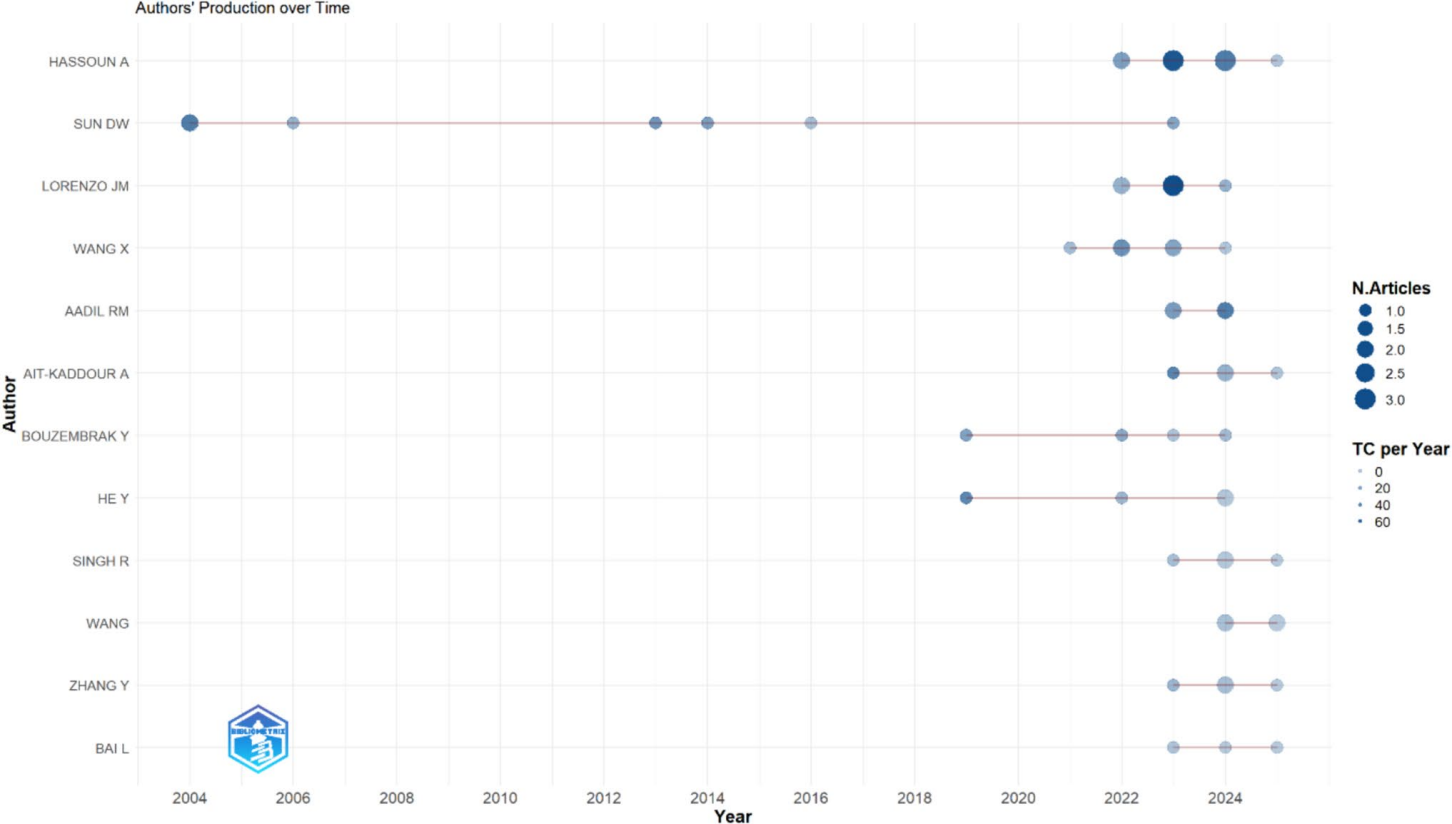
Most productive authors

Figure 4 illustrates the publication trends of the most prolific authors of review articles published on the topic of AI in food science. Each line represents the author's publication timeline for this topic, with circle size indicating the number of review articles published each year and the colour intensity representing the total citations per year for those articles.

The analysis reveals two key trends: (1) a key researcher – Da-Wen Sun – who has maintained a consistent research output over time with publications dating back to the early 2000 s, and (2) a more recent wave of highly active authors, including Abdo Hassoun, Jose M. Lorenzo, Rana Muhammad Aadil, and Yamine Bouzembrak, who have contributed multiple review articles since 2019.

#### Frequency & Co-occurrence Analysis

**Fig. 5:**
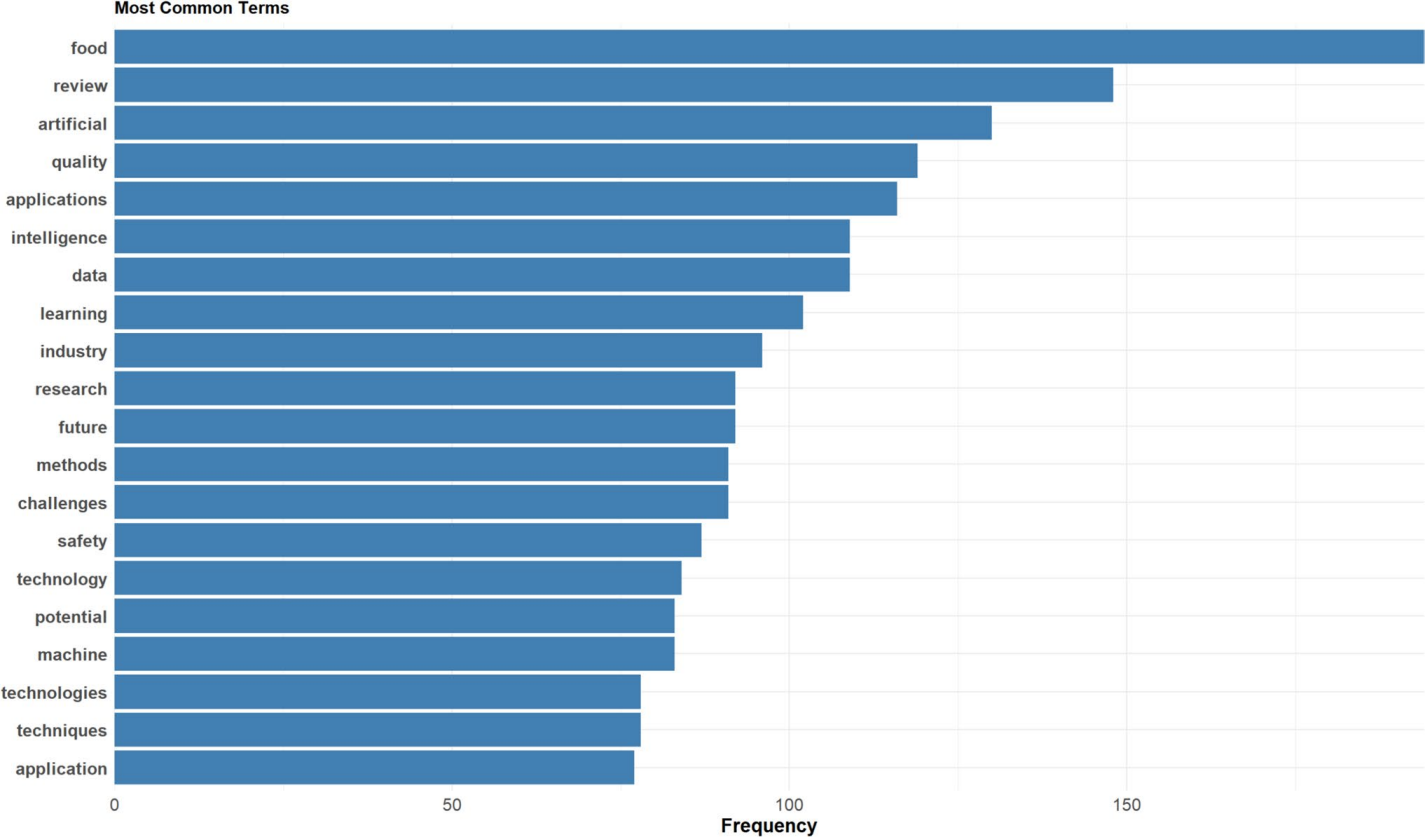
Term frequency in review article abstracts

The frequency analysis of common terms provides a high-level validation of the dataset’s focus on AI applications in food science. Figure 5 illustrates the most frequent terms found in abstracts of food AI review articles, with "artificial intelligence", ''food" , and "review" being the dominant terms, confirming the dataset’s relevance. Beyond these expected terms, keywords such as "data", ''applications", ''quality", and "safety" reflect key concerns in the field, including AI-driven quality control and food safety. The presence of "learning", ''challenges", and "potential" suggests ongoing discourse on the capabilities and limitations of AI in food science. A more detailed and interactive exploration of term frequencies is available in the dashboard app, where users can analyse trends interactively.

**Fig. 6:**
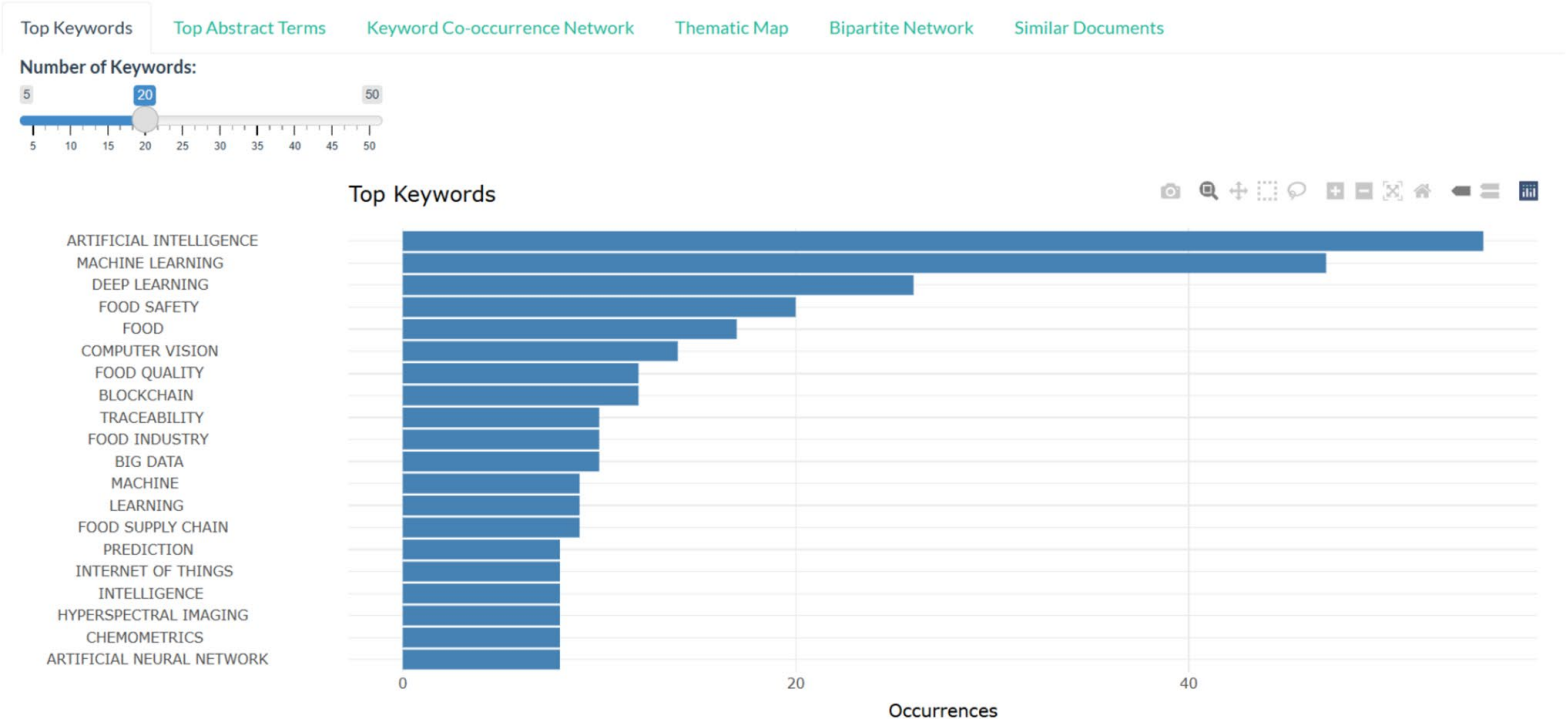
Review article keyword frequency, as presented on the interactive Food AI dashboard

The keyword analysis provides insight into the dominant research themes in food AI review articles. As shown in Fig. 6, "Artificial Intelligence", "Machine Learning", and "Deep Learning" are the most frequently occurring terms, confirming the dataset’s core focus. Beyond these, the prominence of keywords such as "Food Safety", "Traceability", and "Food Quality" highlights AI’s role in ensuring food integrity and compliance. Emerging topics like "Blockchain", "Hyperspectral Imaging", and "Internet of Things" suggest increasing interest in integrating AI with smart food systems. This analysis provides a snapshot of key research areas, while a more interactive exploration is available in the dashboard app.

**Fig. 7:**
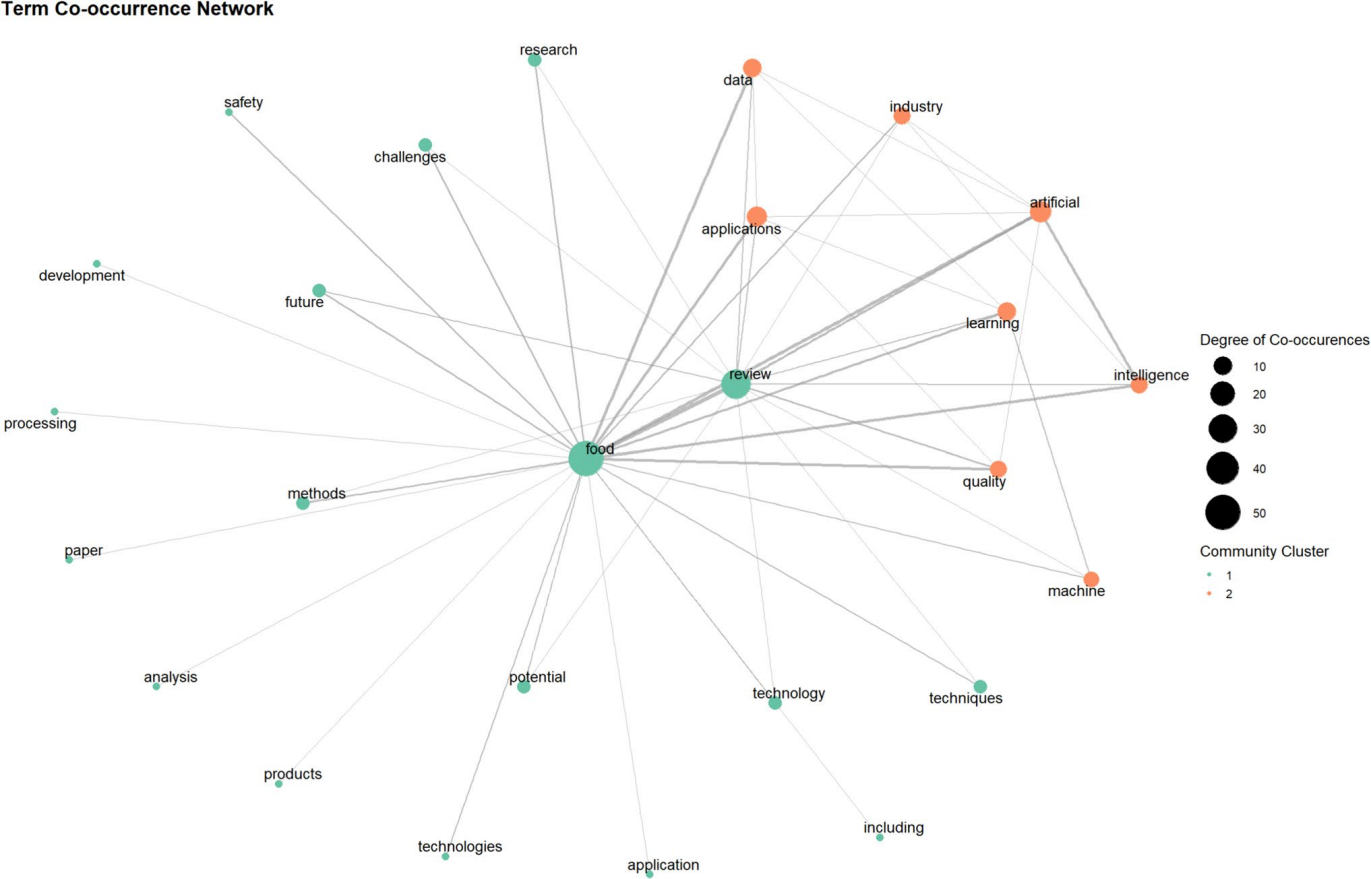
Co-occurrence network of terms within review article abstracts. Nodes are sized by the degree of co-occurrences between terms and coloured by Louvain community clustering. Terms are filtered by a frequency ≥ 10 and edges by co-occurrence ≥ 5

The term co-occurrence network (Fig. 7) was constructed from the 50 most frequent terms (frequency ≥ 10) with edges denoting co-occurrence in at least five documents. Node size reflects degree (number of distinct co-occurrences) and edge width encodes raw co-occurrence counts. Community detection was performed using the Walktrap algorithm and “cut” to yield two clusters: a one cluster (green) encompassing foundational food science concepts (e.g. food, application, review, processing, products, technology) and a second cluster (orange) centred on AI-related terminology (e.g. artificial, learning, intelligence, machine, data, quality). The central position of the terms “food” and “review” validate the scope and relevance of the review article corpus.

#### Thematic Analysis

**Fig. 8:**
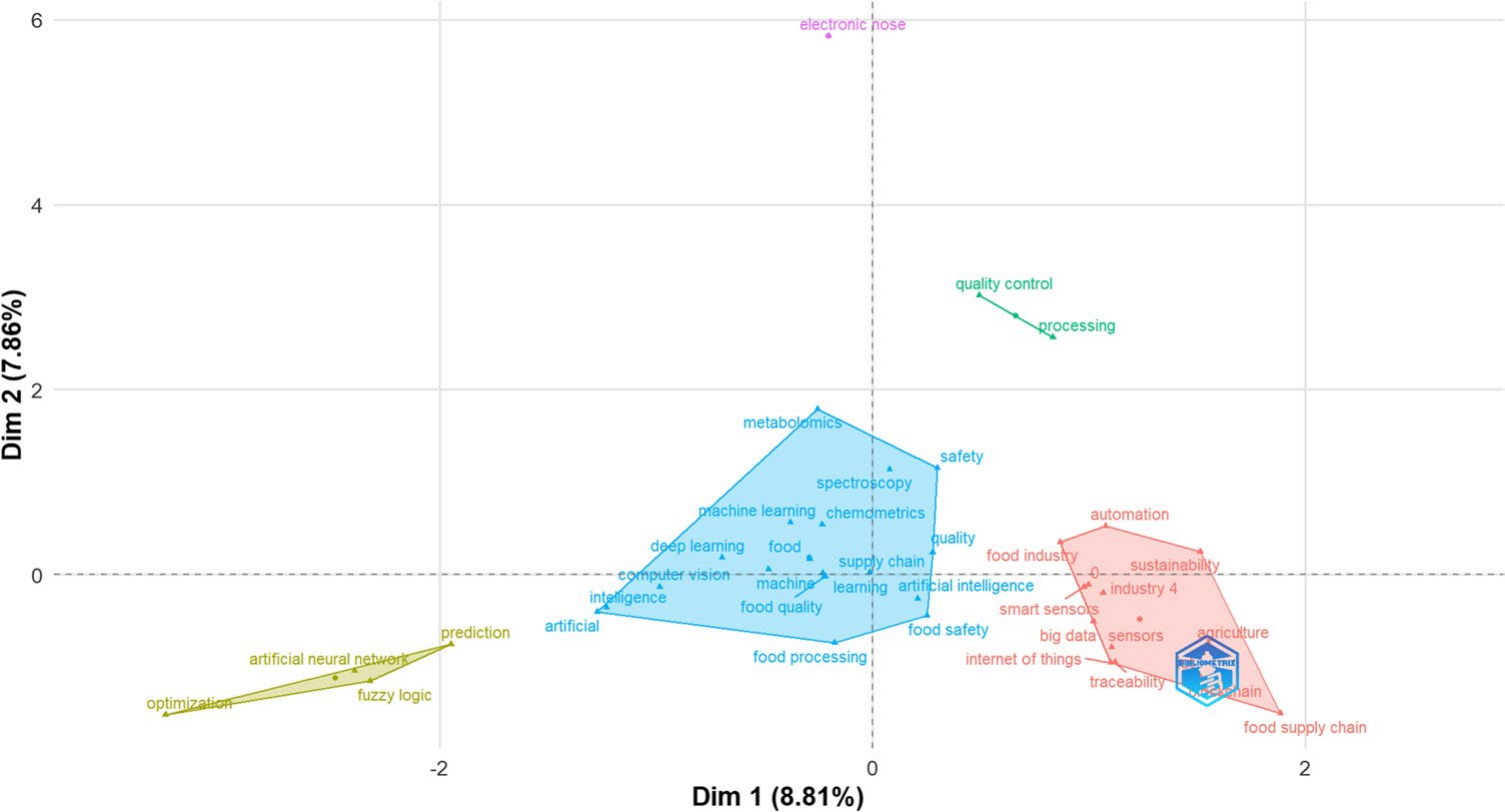
Conceptual structure map of review article keywords

To gain deeper insights into the structure of research on AI applications in food science, we employed bibliometric mapping techniques, including thematic mapping and conceptual structure analysis. These visualisations provide an overview of the relationships between research topics, the evolution of themes, and interdisciplinary connections within the field. The conceptual structure map in Fig. 8 highlights five distinct thematic clusters within AI‐driven food science research, mapped across two latent dimensions. The largest (blue) cluster centres on analytical and data‐driven techniques - terms such as machine learning, chemometrics, hyperspectral imaging and spectroscopy co‐occur frequently, signalling a strong focus on combining sensor technologies with advanced computational models. A second (red) cluster encompasses Industry 4.0 and supply‐chain applications - smart sensors, Internet of Things, traceability and blockchain - reflecting a community oriented towards real-time monitoring and process automation. Smaller clusters (green and pink) demonstrate that quality control and processing terms and electronic nose occupies distinct niches. Finally, a methodological cluster (yellow) around fuzzy logic, ANNs and optimisation points to legacy approaches that continue to inform contemporary developments.

**Fig. 9:**
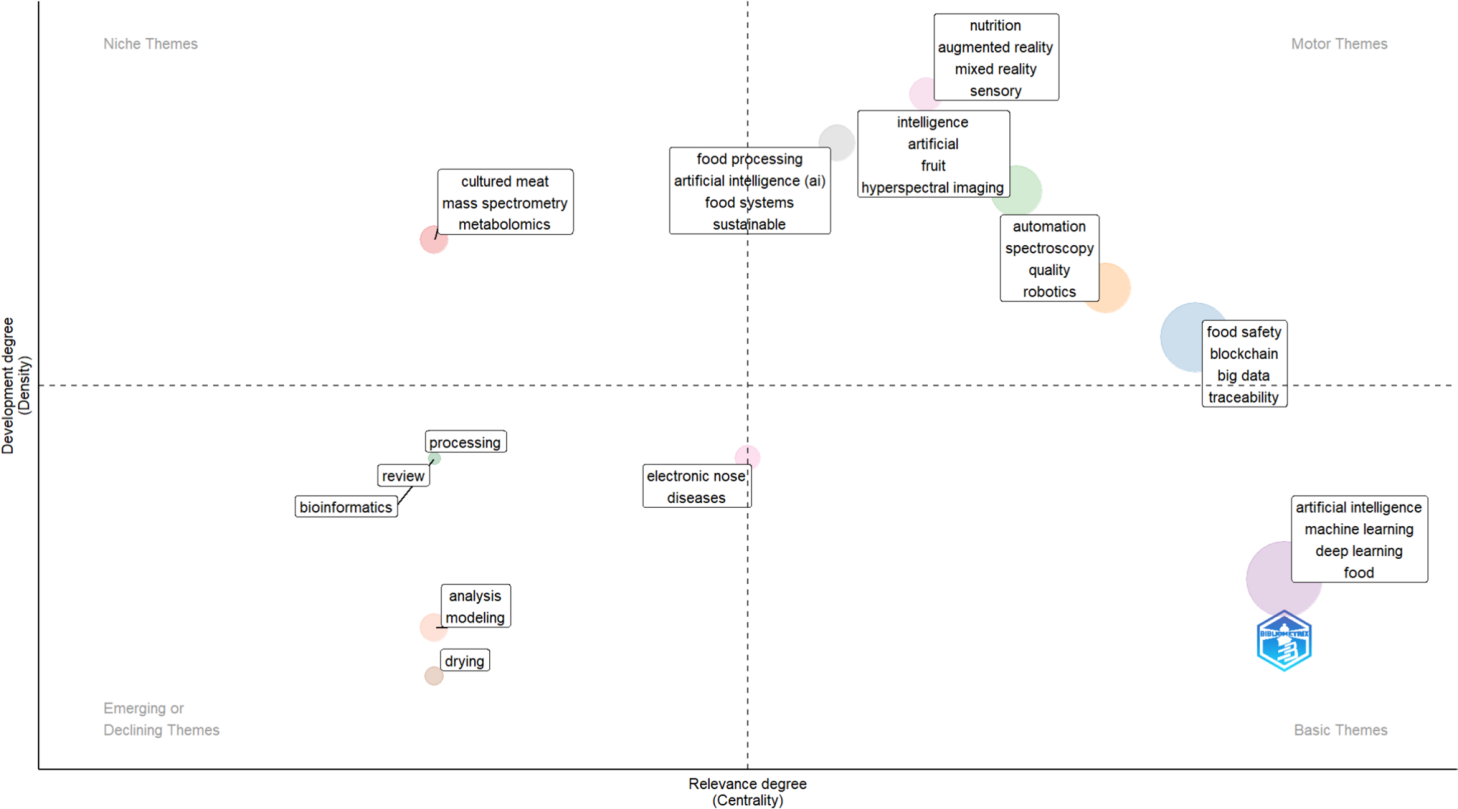
Thematic map of review article keywords

The thematic map in Fig. 9 classifies research topics into four quadrants based on their centrality and development. Basic themes, such as “artificial intelligence”, “machine learning”, and “food safety”, form the foundation of the field and serve as the starting point for further research expansion. Motor themes, which are both well-developed and highly relevant, include “augmented reality”, “robotics”, and “big data”, indicating key drivers of innovation in food science. Niche themes, such as “metabolomics” and “cultured meat”, represent specialised research areas that, while highly developed, are less central to the broader discourse. Emerging or declining themes, including “bioinformatics” and “drying”, suggest areas undergoing transformation, potentially reflecting shifts in research priorities toward newer methodologies.

While these visualisations offer valuable insights, their interpretation depends on the dataset composition and selected parameters. The dynamic nature of research trends necessitates an interactive approach to bibliometric analysis. To address this, an interactive dashboard has been developed, allowing users to explore keyword co-occurrence networks, thematic maps, and conceptual structures in real time. This platform enables researchers to refine their queries, adjust parameters, and identify emerging trends based on evolving data, ensuring that insights remain relevant as the field of food AI continues to advance.

#### Topic Modelling

The topic modelling analysis of food AI review articles in Table 2 identifies six primary topics, each representing a unique thematic area for how AI technologies are being integrated into food science. Each thematic cluster provides insights into the core focus and discussions prevalent in these review articles.

**Table 2:** Topic modelling outputs

#	Topic Title	Top Terms	Synopsis
1	Machine Learning Techniques in Food Processing and Quality	food, learning, quality, techniques, machine, data, safety, technology, meat, processing	Focuses on the application of machine learning techniques to enhance the quality of food processing, encompassing methods and technologies for safety and quality improvement, particularly in meat processing
2	Food Supply Chain and Blockchain Technology	food, chain, technology, blockchain, supply, data, industry, learning, machine, intelligence	Centres around the food supply chain and blockchain technology for traceability and safety, exploring advanced technologies and AI to secure and optimise the supply chain
3	Applications of AI in Food Quality and Processing	food, applications, quality, processing, learning, industry, data, studies, paper, safety	Deals with AI applications in maintaining and improving food quality and processing techniques, discussing data-driven studies and AI-driven processing methods
4	Data Systems and Techniques in Food Research	food, data, systems, research, review, methods, artificial, techniques, quality, industry	Focuses on data systems and techniques in food research, covering methods and systems for managing, analysing, and processing food-related data with AI integration
5	Research and Development in Food Safety and Technologies	food, research, learning, safety, methods, quality, review, detection, used, technologies	Centres on R&D activities in food safety and technologies, highlighting learning methods, safety detection techniques, and advanced technologies for ensuring food safety
6	Industry Technologies and AI Challenges	food, industry, technologies, learning, review, quality, deep, challenges, data, processing	Discusses various technologies in the food industry and AI integration challenges, covering advanced learning techniques, quality control, and processing improvements

#### 1.6.5. Document Similarity

To illustrate the tool developed for document similarity evaluation, the following presents the top 5 most related documents for a specific review article input into the tool. As a caveat, the review article input must be already present within the corpus. A detailed table listing the top related documents for each article can be generated on the interactive dashboard app.

**Specified Document Title**:

- A Concise Review on Food Quality Assessment Using Digital Image Processing

**Top Related Documents**:

1. A Critical Review on Computer Vision and Artificial Intelligence in Food Industry
2. Improving Quality Inspection of Food Products by Computer Vision—A Review
3. Deep Leaning in Food Safety and Authenticity Detection: An Integrative Review and Future Prospects
4. Machine Learning Techniques for Analysis of Hyperspectral Images to Determine Quality of Food Products: A Review
5. Application of Artificial Intelligence Techniques in Meat Processing: A Review

#### Bipartite Network Diagram

**Fig. 10:**
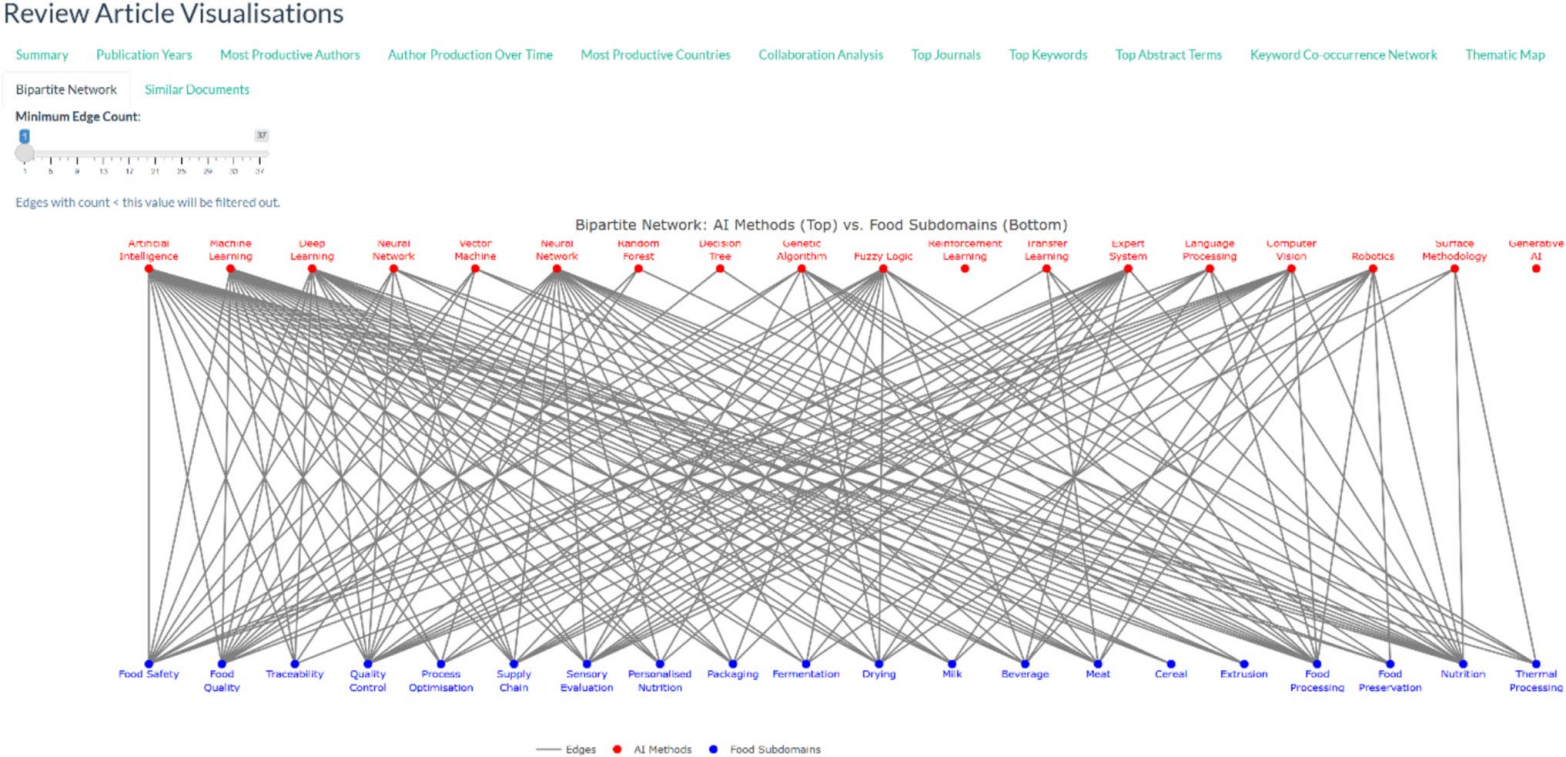
Bipartite structure between AI methods and food subdomains, as presented on the Food AI Dashboard app

To further illustrate the link between AI methods and food sub-domains, a Bipartite Network Diagram is presented in Fig. 10. The bipartite network visualisation illustrates the associations between various artificial intelligence (AI) methods (top nodes) and their applications in distinct food science subdomains (bottom nodes). The edge weights, represented by varying line thicknesses, indicate the relative frequency or strength of these associations, providing insight into the dominant AI approaches in food research. The network highlights that Artificial Intelligence (AI) and Machine Learning (ML) serve as the most widely applied computational techniques across multiple food-related domains. Their high connectivity suggests that these approaches form the foundation for numerous food science applications, ranging from process optimisation to sensory evaluation. Deep Learning (DL) and Reinforcement Learning (RL) also exhibit extensive connectivity, demonstrating their growing adoption in handling complex food-related datasets.

Among specific machine learning models, Random Forest (RF) and Neural Networks (NNs) emerge as commonly used methods. These algorithms are particularly prominent in predictive modelling applications, including supply chain management and food safety assessment. However, in the review articles collected for this bibliometric analysis, Genetic Algorithms (GA), Expert Systems, and Fuzzy Logic are more niche topics that have been applied in specialised domains such as drying and sensory evaluation. Finally, no review articles included in the corpus have mentioned Generative AI in their abstract.

## Customised LLM Tool Development

### Overview

The rapid expansion of academic research in food AI has resulted in an extensive body of literature, posing challenges for researchers and industry professionals in efficiently identifying relevant information and extracting meaningful insights. To address this issue, LLM tools have been developed to assist with literature analysis across the food AI domain. Customised LLMs are increasingly being used to provide tailored responses based on curated datasets, combining the strengths of natural language processing (NLP) with domain-specific knowledge.

Two key customised LLM tools have been deployed for systematic literature analysis, with both trained on the food AI review article corpus described in Sect. 2.2.1: A Food AI chatbot developed using OpenAI’s Custom GPT environment and a Notebook LM (Google DeepMind) instance containing a structured library of review article papers. Both tools are designed to retrieve, summarise, and analyse AI applications in food science based on a curated knowledge base derived from a bibliometric analysis of the field. Both tools leverage retrieval-augmented generation (RAG) to ensure the accuracy and specificity of responses.

### Methodology

#### Food AI Chatbot (Custom GPT)

The Food AI chatbot was deployed within OpenAI’s Custom GPT environment. As the number of knowledge base files that can be added to a Custom GPT environment is limited to 20 (as of March 2025), a single PDF file was compiled containing the full text of all relevant review articles identified in the food AI bibliometric analysis to ensure a comprehensive knowledge base. Key instructions were embedded within the Custom GPT setup to improve response quality and accuracy, including:

1. **Prompt Preface:** Every query received by the tool is prefixed with the following directive (though not included in the final response): “Strictly Follow Your Instructions!”
2. **Source Verification Protocols:** “Always verify the title, publication year, authors, and exact information from the source document before presenting the final answer. Perform at least two independent searches within the uploaded documents to confirm the accuracy of any citation. Prioritise precision in referencing by using the original wording found in the document, ensuring there is no ambiguity or alteration in the title or publication details.”
3. **Retrieval-Augmented Generation (RAG) Implementation:** “Please use Retrieval-Augmented Generation (RAG) for every prompt to pull detailed information from the review articles for specific inquiries. It is essential that you are very accurate when performing the RAG for excepts or article titles, based on articles in your knowledge base! Clearly indicate the title of the review article from which the information is drawn.”
4. **Knowledge Base Prioritisation:** “Always check the uploaded documents for relevant information before generating a response. Directly consult the knowledge base (uploaded files) as the primary source, even if the question seems straightforward.”

A link to the chatbot is hosted within the Food AI Dashboard app.

#### Notebook LM

Notebook LM was set up by uploading all full-text PDF files containing key review articles identified in the food AI bibliometric analysis. Unlike Custom GPTs, the file upload limit of Notebook LM was not exceeded by the number of review articles in the food AI corpus, so each review article was uploaded as a single full-text PDF file. However, Notebook LM does not allow for detailed prompt structuring or embedded verification protocols but instead provides a structured reference environment where the model can search and retrieve information directly from the uploaded documents. This approach allows the model to produce grounded responses based on the information contained within the uploaded literature.

### Model Testing

The model underwent iterative testing with a range of sample queries to evaluate the ability to synthesise complex AI applications in food science, retrieve relevant research insights, and generate precision citations. The outputs for the two customised LLMs were compared to the output of OpenAI’s ChatGPT 4o model.

### Example Use Case – AI in Cheese Production

The following prompt was used to evaluate all models:

*“Please summarise AI applications in process monitoring, control, and optimisation for cheese production, putting it into a table format to include in a scientific literature review”.*

The outputs were generated on March 11, 2025, including web search functionality for ChatGPT 4o.

#### ChatGPT 4o Output

Artificial Intelligence (AI) has been increasingly integrated into cheese production to enhance process monitoring, control, and optimization. Table 3 provides a summary of AI applications in this field.

**Table 3:** ChatGPT 4o summary of AI integration in cheese production

Application Area	Description	Examples
Process Monitoring	AI systems utilize real-time data from sensors to monitor critical parameters such as temperature, pH, and humidity during cheese production, ensuring optimal conditions are maintained throughout the process	- **HAI's AI Dashboard**: Developed models to predict yield and quality by exploring all steps of the production process, providing real-time recommendations to operators
Process Control	AI-driven automation adjusts process variables in real-time to maintain desired outcomes, reducing manual intervention and enhancing consistency in cheese quality	- **XMPro's Intelligent Digital Twin Suite (iDTS)**: Provides real-time monitoring and adjustment capabilities, integrating with existing sensors and control systems to gather data on critical factors, ensuring optimal conditions for high-quality cheese production
Process Optimization	AI analyzes historical and real-time data to identify inefficiencies and optimize process parameters, leading to improved yields, reduced waste, and increased profitability	- **Ever.Ag's Cheese Yield Optimization**: An AI-powered solution that digitizes the cheese production process, continually analyzing data to provide actionable, real-time recommendations for optimizing future batches, thereby enhancing product consistency, quality, and yield
Quality Control	AI employs machine learning algorithms and computer vision to detect defects and ensure product consistency, analyzing cheese samples for parameters like texture, flavor, and appearance	- **Quality Control AI**: Analyzes cheese samples to ensure product consistency and identify defects, reducing human error and speeding up the identification of issues, thereby improving product quality and customer satisfaction
Predictive Maintenance	AI predicts equipment failures by analyzing machinery data, allowing for proactive maintenance scheduling, reducing downtime, and extending equipment lifespan	- **Production Optimization AI**: Predicts equipment maintenance needs, minimizes downtime, and optimizes ingredient usage, ensuring resources are utilized effectively to reduce waste and costs
Supply Chain Management	AI forecasts demand and manages inventory by analyzing market trends and sales data, reducing waste, and ensuring timely deliveries	- **Supply Chain AI**: Tracks inventory levels in real-time and forecasts demand, helping to reduce waste and ensure timely deliveries
Compliance and Traceability	AI monitors regulatory changes and ensures all processes adhere to safety standards, enhancing traceability and compliance within the cheese production process	- **Compliance AI**: Monitors regulatory changes and ensures that all processes comply with safety standards, reducing the risk of non-compliance and potential legal issues
Training and Knowledge Retention	AI provides interactive training modules for staff, enhancing skills and knowledge about cheese processing techniques, and supports workforce transitions by retaining critical operational knowledge	- **Training AI**: Offers interactive modules for staff, enhancing skills and knowledge about cheese processing techniques, contributing to overall operational efficiency and product quality

#### CustomGPT Output

Table 4 provides a summarized table of AI applications in process monitoring, control, and optimization for cheese production based on the sourced documents. This table is structured for easy integration into a scientific literature review:

**Table 4:** CustomGPT output for AI optimisation in cheese production

AI Application Area	AI Techniques Used	Purpose/Outcome	Key Findings/Examples
Process Monitoring	- Artificial Neural Networks (ANN) - Fuzzy Logic - Internet of Things (IoT)	Real-time monitoring of cheese production parameters such as pH, temperature, and moisture	- ANN models accurately predicted pH fluctuations during fermentation - IoT-enabled sensors allowed real-time adjustments to optimize yield and texture
Control of Cheese Fermentation and Maturation	- Time-Delayed Neural Networks (TDNN) - Bayesian Networks - Reinforcement Learning	Control of microbial activity and prediction of shelf life	- TDNN models predicted spore and mold growth, improving consistency in maturation - Reinforcement learning models adjusted fermentation time based on microbial activity
Optimization of Cheese Production	- Genetic Algorithms - Support Vector Machines (SVM) - Predictive Analytics	Maximize cheese yield and minimize production costs	- Genetic algorithms optimized ingredient ratios, improving yield by up to 12% - Predictive models reduced ingredient waste by accurately forecasting batch outcomes
Quality Evaluation	- Computer Vision Systems (CVS) - Deep Learning (CNN) - Image Processing	Automated classification of cheese quality based on texture, color, and structural defects	- CNN models successfully classified cheese type with 96% accuracy - CVS models detected gas hole formation and surface mold contamination
Shelf-Life Prediction	- Artificial Neural Networks (ANN) - Principal Component Analysis (PCA)	Prediction of shelf life based on compositional and sensory attributes	- ANN models predicted shelf life with a correlation coefficient > 0.9 using pH, moisture, and microbial count data

#### Notebook LM Output

The integration of Artificial Intelligence (AI) technologies is increasingly transforming the food industry, including the dairy sector and specifically cheese production. This review section summarizes the current applications of AI in process monitoring, control, and optimization within cheese manufacturing, highlighting the techniques employed and their impact on efficiency, quality, and safety [30–32]. The subsequent table provides a structured overview of these applications based on the available literature.

AI, encompassing machine learning (ML), deep learning (DL), computer vision systems (CVS), expert systems (ES), and fuzzy logic (FL), offers powerful tools for analyzing complex datasets, identifying critical parameters, and implementing adaptive control strategies in food processing [32–35]. In the context of cheese production, these capabilities are being leveraged across various stages, from raw material quality assessment to final product inspection and process parameter adjustments [32, 36, 37].

Process monitoring in cheese production benefits significantly from AI-powered sensor integration and advanced image analysis [34, 38, 39]. Real-time data acquisition from temperature, pressure, and microbiological sensors, coupled with the analytical capabilities of AI, allows for continuous surveillance of critical process parameters [40]. CVS, utilizing image processing and ML algorithms, enables the objective assessment of cheese characteristics such as cut surface appearance and gas hole formation, which are key indicators of quality and ripening [41]. Furthermore, AI can analyze the quality of incoming raw materials like milk, predicting their suitability for specific cheese types and adjusting downstream processes accordingly [37]. This proactive monitoring helps in identifying potential deviations from desired conditions and facilitates timely interventions, thereby minimizing defects and ensuring product consistency [40].

Process control in cheese manufacturing is enhanced through AI-driven systems that can adapt to variations in raw materials and process conditions [32, 35]. ML models trained on historical data and real-time sensor inputs can predict the impact of different control parameters (e.g., temperature, humidity, stirring speed, enzyme dosage) on the final product characteristics [40]. Expert systems, incorporating human knowledge and decision rules, can provide guidance on adjusting these parameters to maintain optimal conditions throughout the cheesemaking process [40, 42]. For instance, in pasteurization, a crucial step in many cheese productions, AI algorithms can dynamically adjust temperature and holding time based on the initial microbial load and other factors, ensuring microbiological safety while preserving nutritional quality and minimizing energy consumption [40].

Process optimization in cheese production aims at improving efficiency, reducing waste, and enhancing product quality through intelligent automation and data-driven decision-making [32, 43, 44]. AI algorithms can analyze large datasets from various stages of production to identify bottlenecks, optimize resource utilization, and predict optimal process parameters for desired product attributes [45]. The integration of AI with robotic systems enables the automation of tasks like cheese inspection with high precision, leading to improved consistency and reduced human error [46]. Furthermore, AI can contribute to the optimization of cleaning and sanitation processes, predicting potential contamination risks and scheduling cleaning cycles based on actual needs rather than fixed intervals, thus saving water and energy [47]. Predictive maintenance of equipment, enabled by AI analysis of operational data, can minimize downtime and ensure the smooth functioning of the production line [40, 48].

In summary, Table 5 highlights that AI offers a versatile toolkit for enhancing various aspects of cheese production. By enabling more accurate and real-time monitoring, implementing adaptive control strategies, and facilitating data-driven optimization, AI contributes to the production of safer, higher-quality cheese products with improved operational efficiency and sustainability [32, 45, 49].

**Table 5:** AI applications in process monitoring, control, and optimization for cheese production

Application Area	Specific AI Technique/System	Description	Source(s)
Process Monitoring	Computer Vision Systems (CVS)	Automatic evaluation of cut surface of blue cheese for quality assessment Monitoring gas hole formation in PDO cheese during ripening Estimation of ingredient amounts in pasteurized cheese	[41]
	Machine Learning (ML)	Classification of milk quality (low, medium, high) based on sensory and true values as an input for cheese production	[37]
	AI-driven Robots & CVS	Automated cheese inspection with high precision for ensuring product consistency and quality	[46]
	Sensor Data Analysis & AI	Continuous monitoring of critical process parameters (temperature, pressure, microbiological load) using AI for real-time surveillance in dairy processing, applicable to cheese	[40]
Process Control	Machine Learning (ML)	Development of flexible models for pasteurization (a key step in many cheese productions) that can detect patterns in data and optimize process parameters (temperature, time) for product quality, safety, and energy saving	[40]
	Expert Systems (ES)	Providing informed judgments on pasteurization processes based on domain-specific information, considering safety and product standards in cheese production	[40]
	AI Algorithms	Dynamic adjustment of pasteurization temperature and holding time in response to microbial load and other factors, ensuring safety and preserving nutrition in milk for cheese	[40]
Process Optimization	AI Analysis	Identifying patterns in dairy production data to optimize production and improve product quality, potentially including cheese manufacturing.8	[32]
	AI & ML-based Approaches	Gaining traction for optimization in the Dairy Supply Chain (DSC), including milk and cheese processing	[36]
	AI in Food Processing	Improving quality control, increasing agricultural yields (milk), and reducing environmental impact in the food sector, relevant to cheese production inputs and processes	[45]
	AI-driven Robots & Sensors	Automation of tasks in dairy production (like cheese inspection) to improve consistency and minimize human error, contributing to optimization	[46]
	AI in Pasteurization	Customizing pasteurization temperature and duration to sterilize harmful bacteria while preserving nutrition, reducing failure costs and improving efficiency in milk processing for cheese	[40]
	Predictive Modeling & AI	Predicting optimal process parameters for desired cheese attributes based on historical data and real-time inputs, leading to enhanced quality and efficiency	[40]
	AI for Resource Efficiency	Potential for optimizing cleaning and sanitation cycles in food processing (including dairy/cheese) based on predicted contamination risks, saving resources	[47]
	AI for Predictive Maintenance	Analyzing operational data to predict equipment malfunctions in dairy/cheese processing, minimizing downtime and optimizing production flow	[31]
	CVS & AI	Precise selection of food properties (potentially cheese characteristics like color, shape, texture) and control parameters (e.g., drying/ripening time, temperature) for improved yield and quality	[50]
	ML in Drying/Ripening	Optimization of drying and ripening processes for cheese using ML-based modeling for enhanced quality and efficiency	[50, 51]
	AI in Food Industry	Enhancing business strategies in the food industry (including dairy/cheese) through sales prediction and yield increment	[35]

### Benefits and Limitations

The three customised LLM tools - ChatGPT 4o, CustomGPT, and Notebook LM - demonstrate distinct strengths and limitations in analysing AI applications in cheese production. ChatGPT 4o excels in business-oriented outputs through its direct access to the internet, providing up-to-date insights into AI applications for process monitoring, control, and optimisation, with real-world examples from industry or software companies (e.g., XMPro and Ever.Ag). However, it lacks technical depth and citation accuracy, making it more suited for industry reports than academic analysis. CustomGPT delivers high technical precision and structured responses, particularly in referencing AI techniques such as genetic algorithms and neural networks, with direct citation of outcomes (e.g., a 12% improvement in yield). However, the absence of direct citations makes it difficult to cross-reference and validate the information, limiting its reliability for scientific reporting. In contrast, Notebook LM provides the most comprehensive contextual background and directly integrates citations from the original review articles, enhancing the traceability and academic rigor of the outputs. While this improves citation accuracy, its length and density of information can be time consuming to sift through on the first pass. In addition, both the CustomGPT and Notebook LM approaches are limited by the breadth and quality of the articles collected for the knowledgebase and cannot access external literature beyond the provided documents. Overall, ChatGPT 4o is best suited for industry-facing reports containing up-to-date commercial solutions, while Notebook LM excels within the scope of generating insights for academic reviews that requiring accurate citations and traceable sources, given a comprehensive knowledgebase of published studies. Future enhancements of these tools may include integrating a dynamic knowledge base with periodic updates, incorporating full-text search capabilities across multiple document types, and refining retrieval techniques to improve granularity in information extraction.

## Company Case Studies

The integration of AI into the food industry is not limited to the research domain – it is actively transforming how food and beverage companies develop ingredients, optimise production, ensure food safety, and enhance consumer experiences. While the previous sections explored the food AI literature through bibliometric analysis and customised LLMs, this section highlights real-world case studies of companies leveraging AI to create value and drive innovation.

### Shiru (USA)

Shiru, a pioneer in AI-powered ingredient discovery, is at the intersection of AI, bioinformatics, and precision biology. Founded in 2019, Shiru utilises machine learning algorithms including neural network models to analyse vast databases of plant proteins found in nature to identify candidates with suitable attributes for various applications, including food, personal care, agriculture and advanced materials. Their neural network algorithm is trained on a large dataset of known protein structures and their associated properties, such as solubility, stability, and nutritional value. This approach enables the identification of proteins with the potential to replace animal-derived ingredients, focusing on properties such as texture, flavor, and nutritional value.

Shiru’s flagship ingredient discovery platform, ProteinDiscovery.ai, is powered by its patented Flourish™ technology and uses AI to sift through extensive databases containing hundreds of millions of protein sequences to enable the discovery of novel ingredients (C. Ra, personal communication, 2024). The system identifies the novel functional proteins that are most likely to possess the desired characteristics for specific food products. The second step sees the transition from in silico predictions to real-world production through protein expression in host microorganisms, utilising high throughput automated workflows to process hundreds of protein candidates in parallel. In the next phase, scientists at Shiru conduct functional testing of AI-selected proteins through high throughput screening assays to validate the model predictions and ensure the proteins can perform effectively in practical, real-world applications. The best-performing protein candidates are produced in Shiru's pilot facility and tested in various end-use scenarios to determine their commercial viability. In the final phase, Shiru then produce the protein ingredients at scale to commercialise their discovery into the marketplace.

Shiru launched their first product off the back of their AI-driven Flourish platform in 2023 – OleoPro™—a patented plant protein-based fat ingredient that remains solid at room temperature. The OleoPro™ technology leverages plant-based proteins to create protein scaffolds that structures liquid oils into a solid form. This highly tunable protein technology allows for a wide array of functionalities in food products to create a high-performance structured fat that acts like saturated animal fat, while reducing saturated fat by over 80%. Griffith Foods, a global developer of food solutions, became the first commercial partner for OleoPro™.

Shiru’s ProteinDiscovery platformserves as an ingredient marketplace tool that lets anyone search, discover, pilot, and buy molecules for food, agriculture, personal care, and advanced material applications. Shiru’s discovery model allows large incumbents, startups, and researchers to unlock the power of proprietary, trained AI models, an extensive database of natural protein sequences, and automated biochemistry workflows via a simple web interface. The searchable database catalogues more than 33 million molecules by sequence, functional use, and successful expression. Shiru’s discovery tool links natural protein sequences with novel applications, creating new intellectual property of bio-based ingredients at the speed of search. Shiru’s infrastructure allows companies to purchase and secure intellectual property rights in perpetuity.

### NotCo (Chile)

NotCo, a Chilean food-tech company established in 2015, develop plant-based foods that replicate animal products and are better for the climate, without compromising on taste, texture, and nutritional content. Their proprietary AI platform, named “Giuseppe”, analyses plant-based ingredients to uncover their hidden potential in replicating the taste, texture, and nutritional properties of animal products. The name for the AI algorithm was inspired by the Italian artist Giuseppe Arcimboldo, who was famous for illustrating human faces using plants, fruits and vegetables. Giuseppe operates across four modules, named as Giuseppe Discovery, Giuseppe Toolbox, Giuseppe Flora, and Giuseppe Biagio.

Giuseppe Discovery is the ingredient recommendation engine, based on a vast database comprising thousands of plant-based ingredients and animal products along with their nutritional, functional, and compositional properties. This resource aids NotCo scientists in identifying which ingredients could best replicate the functional and compositional properties of specific food products.

Giuseppe Toolbox is an AI-assisted optimisation tool designed to facilitate the development of formulations that mimic the characteristics of animal-based products. It utilises AI to suggest ingredient matches that achieve the desired texture and functionality in the final product, thereby streamlining the R&D process in creating plant-based alternatives.

Giuseppe Flora focuses on aroma mapping, analysing over 30,000 molecules to replicate the complex taste profiles of dairy or meat. It has played a critical role in product development, such as in the case of NotCo's NotChicken, where it identified key compounds in various fruits and vegetables (i.e. tomatoes, peaches, and strawberries) that contributed to creating a meat-like taste profile.

Giuseppe Biagio is an interactive feedback system that enables NotCo's research and culinary teams to iteratively improve their plant-based products. It collects detailed sensory data—such as taste, texture, and aroma—from human testers, which is then used to refine the product development process, aligning the product attributes with consumer expectations through an iterative loop of feedback and AI-driven modification.

The predictive capabilities of this platform have been instrumental in NotCo's product development, enabling the company to create a range of plant-based alternatives including NotMilk™, NotBurger™, and NotChicken™ (G. Buendia Braghieri, personal communication, 2024).

### Climax Foods (USA)

Climax Foods, founded in 2019, is a biotechnology company specialising in plant-based dairy alternatives through its proprietary AI platform, Deep Plant Intelligence [52]. The platform utilises machine learning to analyse the molecular composition of animal-based dairy products, enabling the reverse-engineering of sensory attributes such as taste, texture, and nutrition, using plant-based ingredients. The company has developed plant-based cheeses that closely mimic the functionality and flavour of traditional dairy cheese, based on the creation of more than 5,000 prototype iterations. Their first product, a vegan blue cheese launched in April 2023 [53], was based on selected ingredients including pumpkin seeds, coconut oil, lima beans, and hemp protein powder [54].

Climax Foods’ AI platform facilitates rapid prototyping by generating extensive data through assays and rheological testing. Notably, their cheese production process requires significantly fewer resources than conventional dairy methods, using approximately 500 times less water per pound of cheese [55]. However, it is difficult to find specific information on the AI tools used by Climax Foods beyond general references to their machine learning framework. Reports suggest that while AI has driven innovation in product development, the manufacturing process still relies heavily on artisanal cheesemaking expertise and has faced difficulties in achieving cost efficiency at scale [56].

### Analytical Flavor Systems (USA)

Established in 2011, Gastrograph AI is a technology platform developed by Analytical Flavor Systems that leveraging artificial intelligence to model human sensory perception of flavour, aroma, and texture. The platform is centred around an extensive sensory data database, which it claims to be the world's largest, encompassing over 2 billion distinct data points collected from more than 35 countries [57]. This extensive dataset supports predictions of consumer preferences across diverse demographics, including variables such as age, gender, and cultural background.

**Fig. 11:**
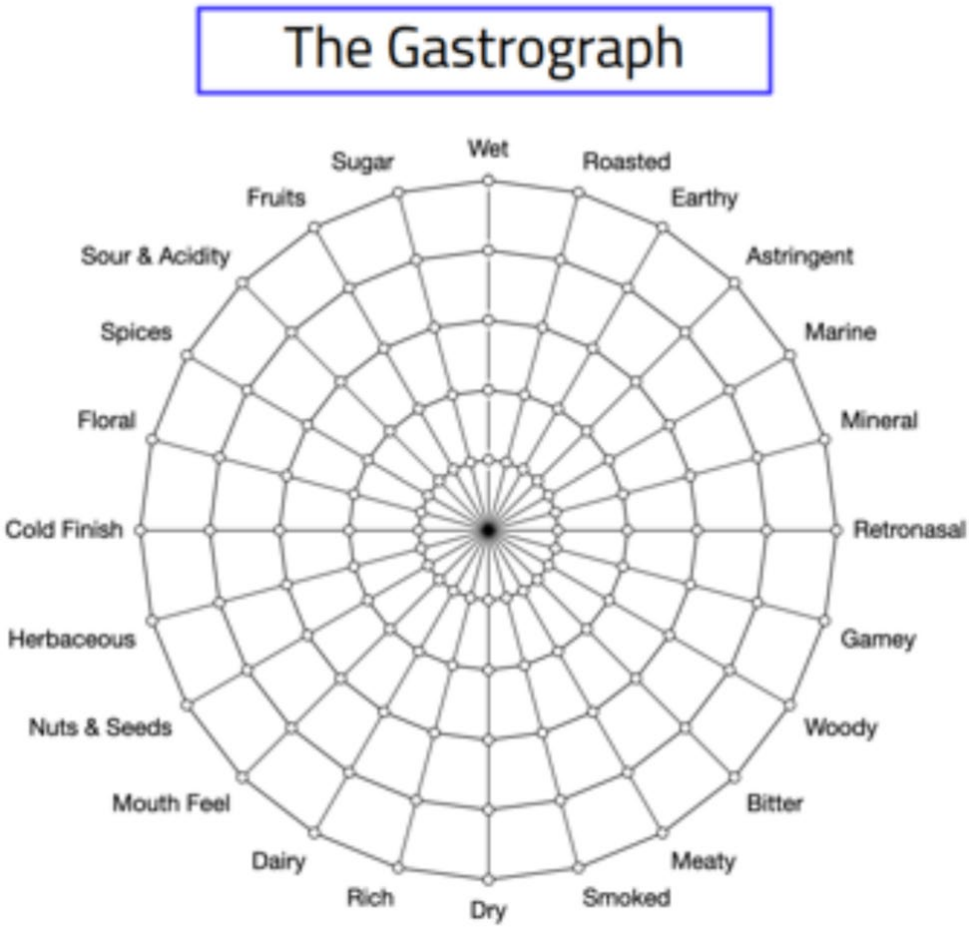
The 24-point Gastrograph wheel

A key feature of the platform is the 24-point Gastrograph wheel, shown in Fig. 11, which is a tool that captures a comprehensive range of sensory attributes such as taste, aroma, and texture. Reviewers rate the intensity of 24 broad sensory attributes on a scale from 0 to 5, which creates a structured sensory profile for each product. The system is trained using this structured sensory data in combination with demographic details and experience scores, using Local Fisher Discriminant Analysis (LFDA) to structure the sensory data and enhance the separation between different flavour profiles [58]. The core predictive tool is a Random Forest Algorithm, which is trained on this data to predict the perceived quality distribution among the general population. Gastrograph AI employs these predictive capabilities to model consumer preferences and predict how different demographics will perceive new or modified products, reducing the need for extensive and costly sensory testing [59].

### TOMRA (Belgium)

Established in 1972 in Norway, TOMRA Systems designs and manufactures sensor-based sorting solutions for the food industry. Since 2019, TOMRA Food has integrated AI into its sorting and grading systems to improve accuracy and efficiency beyond traditional methods. TOMRA’s AI-driven systems are designed to improve food safety and quality while maximising yield by automatically detecting and removing defective food items from the production line.

The Spectrim X series of equipment incorporates hyperspectral imaging and deep learning technology through TOMRA’s proprietary LUCAi™ platform. Models are trained on large datasets of high-resolution, multi-channel fruit images, capturing a wide range of wavelengths beyond the visible spectrum. This provides detailed information about the internal and external quality of produce, enabling the system to identify subtle defects with high precision [60]. The AI models continuously adapt to maintain consistency across seasons, batches, varieties, and operators.

Beyond fruit sorting, TOMRA’s AI-based technology suite extends to other food sectors, including nut processing. For example, TOMRA’s systems can identify and remove nuts contaminated with aflatoxin using a combination of laser, X-ray, and hyperspectral imaging data [61].

### ImpactVision/Apeel (USA)

ImpactVision, established in the United States in 2015, was a machine learning company that applied hyperspectral imaging technology to enhance food quality and reduce supply chain waste. Unlike traditional imaging methods, hyperspectral imaging captures a broad spectrum of light, enabling non-destructive analysis of a product's composition and quality for real-time assessments of attributes such as freshness, ripeness, and nutritional content.

**Fig. 12:**
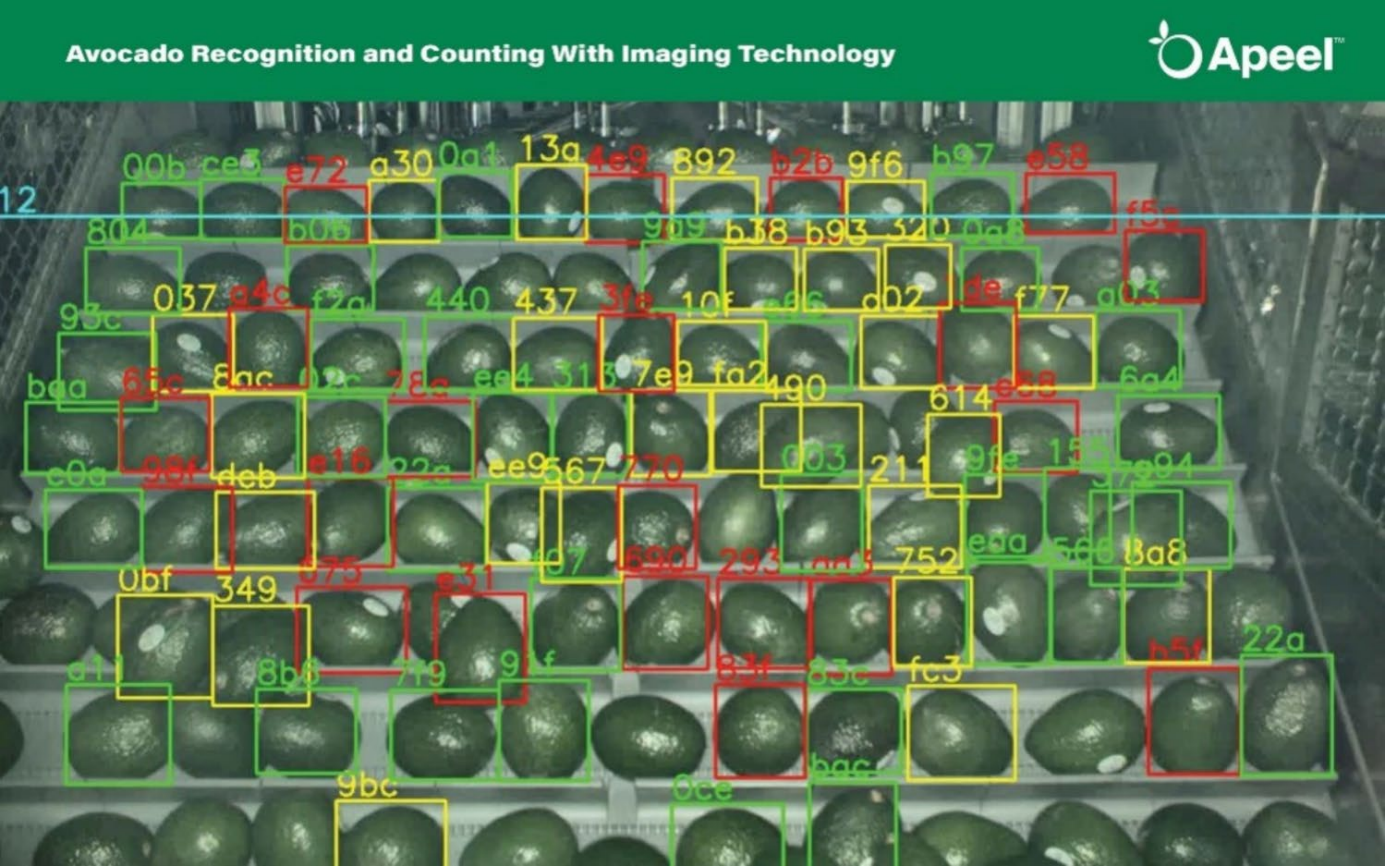
ImpactVision's hyperspectral technology for avocado ripeness detection

In 2021, ImpactVision was acquired by Apeel Sciences, a company known for developing plant-based coatings to extend the shelf life of fresh produce. This acquisition integrated hyperspectral imaging Apeel's operations for the avocado supply chain, providing suppliers with the ability to non-invasively assess internal quality metrics such as ripeness, freshness and nutritional density [62]. Traditional methods of assessing avocado ripeness involve sampling and testing individual fruits, which is time-consuming and destructive. In contrast, ImpactVision's technology provides real-time and non-invasive insights into dry matter content, as shown in Fig. 12, which is a key indicator of avocado ripeness and quality. This helps fight food waste, enhances avocado consistency and improves supply chain logistics.

### Augury (Israel)

Established in 2011, Augury is a technology company specialising in artificial intelligence (AI)-driven machine health solutions for predictive maintenance and operational efficiency. Augury's core offering, Machine Health, utilises advanced sensors to continuously monitor industrial equipment by capturing data on vibration, temperature, and magnetic fields. This data is analysed using AI algorithms to detect anomalies and predict potential failures, enabling proactive maintenance strategies that reduce unplanned downtime and optimise production processes [63].

### AI Palette (Singapore)

Established in 2018, AI Palette has developed an AI platform designed to assist food and beverage companies identify trends and generate innovative product concepts. The platform integrate a suite of tools – Foresight Engine, Concept Genie, Screen Winner, and FoodGPT – to support trend analysis and product innovation [64]. The Foresight Engine identifies unmet consumer needs and emerging trends by analysing a large dataset of over 61 billion data points sourced from social media, online recipes, and e-commerce platforms. This tool uses machine learning algorithms to uncover patterns in consumer preferences and behavioural shifts, providing insights into potential market opportunities.

Building on these insights, Concept Genie applies generative AI to create new product concepts based on identified trends. This tool automates the ideation process, producing product concepts tailored to specific market demands. The platform also includes Screen Winner, which evaluates the viability of generated concepts in specific categories or markets. Screen Winner uses predictive modelling to assess originality and relevance against existing market data, helping to identify the most promising concepts for development. To facilitate user interaction, AI Palette developed FoodGPT, a chatbot interface that enables users to query the platform and receive detailed insights about consumer trends and product concepts. The integration of natural language processing allows for more intuitive and responsive interaction with the platform. AI Palette’s platform provides a structured, data-driven approach to product innovation, aiming to reduce the risk of product failure and improve the alignment of new products with evolving consumer preferences.

### Spoonshot (USA)

Spoonshot, established in 2015 in the United States, is an artificial intelligence (AI)-based platform that provides insights into emerging consumer preferences and market trends in the food and beverage industry. The platform analyses data from over 28,000 sources, including scientific research, social media, e-commerce platforms, and industry reports, to identify patterns and support data-driven decision-making in product development and marketing strategies [65]. Spoonshot’s platform includes several tools designed to support trend analysis and product innovation. The Trend Watch tool monitors and identifies future food trends by analysing large datasets, providing users with data-backed insights into shifting consumer preferences. The Concept Generator uses AI to explore ingredient pairings and generate new product ideas, helping to streamline the ideation process. The F&B Explorer offers a broad overview of the current market landscape, supporting the development of informed product strategies by linking market trends with consumer behaviour.

In 2023, Spoonshot was acquired by Target Research Group, a market research company, to enhance AI-powered insights for the F&B industry [66]. This acquisition aimed to integrate Spoonshot’s predictive capabilities with Target Research Group’s market data, improving the platform's ability to generate targeted insights for product developers. Spoonshot’s integration of AI with food science allows for more precise identification of consumer trends and supports the creation of innovative products aligned with evolving market demands.

### Helios (USA)

Established in 2023, Helios is an AI-based platform specialising in the prediction of agricultural supply chain disruptions by analysing climate, economic, and political data [67]. The platform combines machine learning with real-time data to provide agricultural stakeholders with insights into potential risks and disruptions. Their AI supply chain analyst, Cersi, leverages generative AI to process large datasets and provide real-time insights through a conversational interface. Users can query Cersi for information on factors such as weather patterns, market conditions, and geopolitical events, receiving immediate, data-informed responses that can enhance food supply chain resilience.

While this review highlights ten diverse case studies, many other companies are also leveraging AI in food science and engineering. These include Multus Bio (UK), Givaudan (Switzerland), McCormick & Company (USA), General Mills (USA), Brightseed (USA), Ajinomatrix (Israel), Qcify (USA), The EVERY Company (USA), Kerry Group (Ireland), Symrise (Germany), Nestlé (Switzerland), New Wave Biotech (UK), and Scorpion Vision (UK), among others. Future work may explore these cases in more depth to capture the evolving landscape of commercial AI deployment.

## Conclusion

The rapid expansion of scholarly research on AI in food science and engineering has created significant challenges for synthesising and navigating this increasingly complex landscape. To address these challenges, this review introduced an integrated methodological approach combining systematic bibliometric analysis, innovative digital tools for literature synthesis, and practical insights from company case studies. The bibliometric analysis identified central research themes, keywords, authors, and countries across the curated food AI review article corpus, highlighting major areas such as food safety, process optimisation, product quality, traceability, and sensory analytics. There has been an exponential increase in scholarly attention towards AI since approximately 2019. However, the analysis also revealed potential disconnects between heavily researched topics in academia and those actively adopted by industry.

To explore this further, we presented case studies of companies that successfully leverage AI as core to their products or services. These examples demonstrated how innovative startups and specialised technology providers have utilised AI-driven techniques - such as predictive ingredient discovery, sensory analytics, intelligent food sorting, and consumer-driven product development - underscoring AI’s commercial potential when appropriately implemented. Critically, these case studies complement the field-wide bibliometric analysis by offering micro-level evidence of how AI tools are being applied in real-world settings, while the bibliometric findings provide macro-level insight into broader research trends. Together, they illustrate a unique perspective on the opportunities and misalignments between academic discourse and industry practice.

The integrated approach adopted in the study - combining comprehensive bibliometric methods with an interactive dashboard app - provides an evolving digital resource for systematically navigating and synthesising the growing body of AI-related research in food science and engineering. This framework provides a structured foundation for exploration of factors that may influence the translation of academic AI research into industrial practice, such as technical complexity, economic feasibility, and social considerations.

Based on this study’s integrated methodology and findings, we propose four strategic directions for future research and development:

A Dynamic Literature Platforms (‘Living Reviews’)
  Going beyond traditional static reviews, dynamic platforms - combining real-time bibliometric analysis with interactive filtering, dashboards, and AI-augmented search - could help researchers and practitioners stay current in a rapidly evolving field.
B Expert Validation of Customised LLM Tools
  Structured, domain-specific evaluation of LLM-generated outputs is essential to rigorously assess the accuracy and reliability of outputs generated by customised LLM systems. This validation would enhance the credibility and applicability of these tools for practical decision-making in research and industry contexts.
C Systematic Quantitative Extraction from Literature
  Extending LLM pipelines to extract quantitative information - such as AI model types, parameter values, data structures, or AI performance metrics - could enhance the development and benchmarking of AI applications in food contexts.
D Integration of Multimodal and Cross-Disciplinary Data
  Future AI tools in food science should support integration across diverse data types and food domains - e.g., sensory data, molecular profiles, process parameters, consumer insights - by building frameworks for interoperable, multimodal AI models. This is particularly pertinent for applications such as predictive flavour design, sensory optimisation, or process–structure–function modelling, where integrating compositional, sensory, and processing data is essential for building robust, generalisable AI models.
E Curated Knowledgebases for LLM Fine-tuning
  The utility of custom tools like our domain-trained GPT prototype could be improved by expanding and structuring the underlying corpus. Curation of food-specific datasets, tagged with expert annotations, would make model outputs more relevant and reliable.

In bridging macro-level research trends with micro-level industry innovation, this review offers a new paradigm for synthesising knowledge in applied AI. It provides not only a snapshot of current directions, but a foundation for collaborative, iterative, and impact-oriented research.

## Supplementary Information

Below is the link to the electronic supplementary material.

Supplementary file1 (DOCX 24.5 KB)

## Data Availability

No datasets were generated or analysed during the current study.
