# Supplementary material for: Mapping the AI Landscape in Food Science and Engineering: A Bibliometric Analysis Enhanced with Interactive Digital Tools and Company Case Studies
Source: Food Eng Rev. 2025 Jul 1;17(3):465–89. doi: 10.1007/s12393-025-09413-w (PMC12494660; doi:10.1007/s12393-025-09413-w)
Supplement: Supplementary file 1 — Supplementary file1 (DOCX 24.5 KB) [file 12393_2025_9413_MOESM1_ESM.docx]

**Online Resource 1: Interactive Dashboard for AI in Food Science & Engineering**

**Overview**

This interactive R Shiny dashboard provides an accessible, structured exploration of Artificial Intelligence (AI) applications in food science and engineering. It draws on a curated database of peer-reviewed review articles, supplemented with targeted analyses from primary research articles, enabling users to identify key trends, contributors, and thematic developments in the field.

**Dashboard Structure**
The dashboard is organised into multiple tabs:

- **Home** – Introduction and contextual background of the review.
- **Methodology** – Details on corpus construction and bibliometric analysis.
- **Food AI Review Database** – A searchable, filterable table of all food AI review articles.
- **Review Article Visualisations** – Interactive plots on publication year trends, author productivity, country distribution, co-occurring keywords, thematic clustering, and more.
- **Research Article Analysis** – Supplementary analysis of research papers cited by the reviews, including keyword frequency plots and word clouds.
- **Custom LLM Tools** – Links to tailored Food AI GPT and NotebookLM interfaces for refined literature interrogation.
- **Image Gallery** – A carousel and grid-style view showcasing AI-related diagrams, taxonomies, and frameworks from the literature.

All plots are interactive (via plotly) or rendered using ggplot2.

**Key Features**

- **Exploratory Visualisations:** Dynamic, interactive charts and network graphs to visualise bibliometric trends and research clusters
- **LLM Integration:** The dashboard links to custom GPT and NotebookLM tools designed to support deeper literature exploration through natural language queries.
- **Visual Repository:** A curated image gallery showcasing figures from the reviewed literature.
- **Filter by Subdomain:** Users can search and filter data for further analysis.

**Access**

The dashboard is available at: <https://pennellsy.shinyapps.io/FoodAI_Review_App/>

**Technical Details**

- **Platform:** Built with R Shiny for a responsive, browser-based interface.
- **Data Sources:** Review articles were sourced from Scopus and Web of Science using AI- and food-related keyword queries.
- **Update Frequency:** Periodically updated with new literature and image contributions.

**Usage Notes:**

- Navigate using the tabbed interface.
- Hover over plots for tooltips, zoom, and filter options.
- For targeted literature queries, use the linked LLM tools provided in the "Custom LLM Tools" tab.
- Feedback is welcomed via the associated review article’s corresponding author contact information.
